# Chronic myocardial and coronary arterial effects of intracoronary supersaturated oxygen therapy in swine with normal and ischemic-reperfused myocardium

**DOI:** 10.1038/s41598-022-09776-8

**Published:** 2022-04-06

**Authors:** Grzegorz L. Kaluza, Jeffrey L. Creech, Ariel Furer, Maxwell E. Afari, Krzysztof Milewski, Geng-Hua Yi, Yanping Cheng, Gerard B. Conditt, Jenn C. McGregor, Donald Blum, Serge D. Rousselle, Juan F. Granada, Daniel Burkhoff

**Affiliations:** 1 Skirball Center for Innovation, Cardiovascular Research Foundation, Orangeburg, NY USA; 2 ZOLL TherOx, Inc., Irvine, CA USA; 3 Sheba Tel HaShomer City of Health, Ramat Gan, Israel; 4 Maine Medical Center, Portland, ME USA; 5 American Heart of Poland, Katowice, Poland; 6 StageBio, Frederick, MD USA

**Keywords:** Cardiology, Cardiac device therapy, Cardiovascular biology, Interventional cardiology

## Abstract

The study assessed chronic myocardial, coronary and systemic effects of intracoronary supersaturated oxygen (SSO_2_) therapy. Left anterior descending coronary arteries of 40 swine were stented and randomized to 90-min selective intracoronary infusion of SSO_2_ (pO_2_ 760–1000 mmHg) or normoxemic saline. In 20 out of 40 animals, SSO_2_ delivery followed a 60-min balloon occlusion to induce myocardial infarction (MI). In both normal and MI models, intracoronary treatment with hyperoxemic SSO_2_ therapy showed no evidence of coronary thrombosis. There were no biologically relevant differences between treatments at either time point in regard to coronary intervention site healing and neointimal growth. No signs of any myocardial or systemic toxicity were observed after 7 or 30 days. A trend was observed toward reduced incidence of microscopic MI scars and reduced infarct size in histopathology, as well as toward better recovery of echocardiographically evaluated global and regional contractility at 30 days. No treatment related infarcts or thromboemboli were observed in the downstream organs.

## Introduction

Myocardial infarction (MI) remains an important and common cause of morbidity and mortality. This is true despite the revolutionary role revascularization as well as secondary prevention efforts have had in the last few decades on patients’ outcomes. Reperfusion by means of percutaneous coronary intervention (PCI) is the recommended revascularization modality for patients with ST-elevation MI. It confers significant mortality benefit and has been shown to reduce the rates of recurrent ischemic events and re-infarction^1^. However, even with a flow of Thrombolysis in Myocardial Infarction (TIMI) grade of 3 at the epicardial level, adequate tissue reperfusion assessed by myocardial perfusion grade has only been achieved in up to 66% of patients^2–4^.

Indeed, myocardial salvage does not depend solely on restoring epicardial reperfusion. Among many different factors, microvascular dysfunction and reperfusion injury are important in the degree of residual myocardial damage, and as such have been the focus of several therapeutic approaches^1^. The use of supersaturated oxygen (SSO_2_) perfusion to the coronary arteries has been shown, definitively, to improve microvascular flow and left ventricular function in animal models of myocardial infarction^5,6^. These were followed by a series of clinical trials eventually leading to its marketing approval for clinical use in the US^7–9^. However, a number of detailed questions related to tissue effects and safety of SSO_2_ therapy have remained. Accordingly, the purpose of this study was to provide a comprehensive assessment of safety (local and systemic) and microscopic myocardial tissue effects of 60 min of intracoronary SSO_2_ therapy 7- and 30-days after administration in normal myocardium and in infarcted myocardium induced by temporary coronary occlusion. In contrast to previous animal studies, the present experiment involved implantation of stents in all coronary artery to also assess whether SSO_2_ has any influence on coronary artery healing or stent endothelialization.

## Methods

### Experimental design

The study was approved by the Institutional Animal Care and Use Committee of the CRF Skirball Center of Innovation, Orangeburg, NY. All animals received standard care outlined in accordance with the Guide to Care and Use of Laboratory Animals by National Academy of Science and Animal Welfare Regulations.

**Figure 1:**
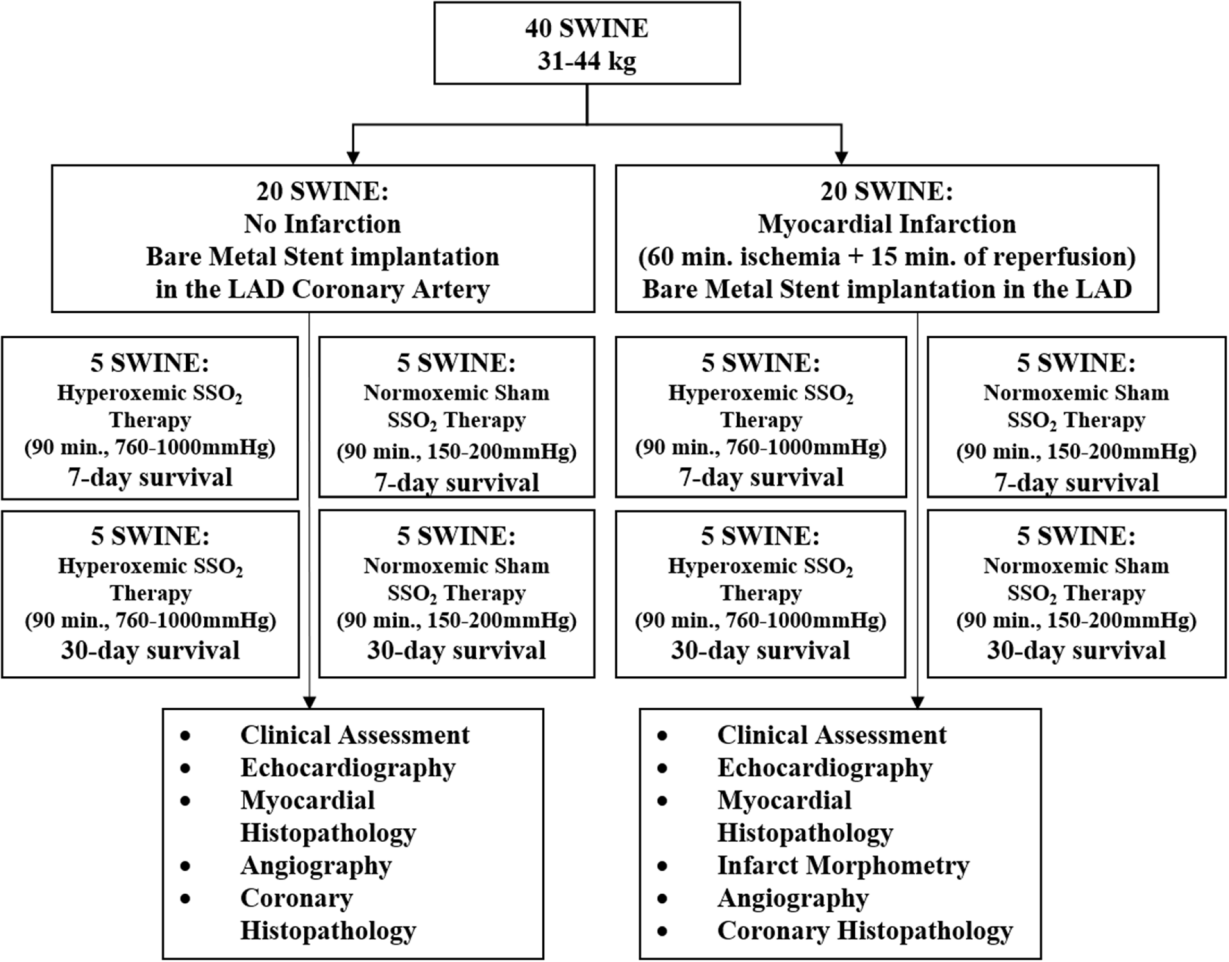
Essentials of study design in form of a flow chart.

A total of 40 domestic swine (mean body weight 39 ± 4 kg) were included in this study. The mean age at the time of inclusion was 4.60 ± 0.49 months. Induction of anesthesia was achieved with a rapid acting general intramuscular anesthetic (tiletamine + zolazepam, 2–5 mg/kg) supplemented by muscarinic anticholinergic (glycopyrrolate, 0.2 mg/ml, and dosage 0.005–0.02 mg/kg). All animals underwent endotracheal intubation and were maintained with a continuous inhalation of 1–3% isoflurane. Arterial femoral access was obtained, under general anesthesia, using a cutdown technique. Anticoagulation with heparin was achieved (3,000–10,000 U) to maintain activated coagulation time ≥ 250 s. Once under anesthesia, baseline transthoracic echocardiograms (TTE) were performed to rule out any pre-existing underlying structural pathology. Following a pre-established matrix (Fig. 1), the swine were randomized to either a MI (7- or 30-day follow-up) group or to a Non-MI (7- or 30-day follow-up) group and subsequently randomized to either SSO_2_ therapy (hyperoxemic therapy) or a control group that received normoxemic reperfusion. All animals received a bare metal stent in the target coronary artery to mimic the clinical scenario of PCI. The essentials of study design are summarized in Fig. 1.

### Model creation: non-MI animals

Using baseline angiography, suitable segments of the left anterior descending coronary artery (LAD) were selected for stent placement. A bare metal stent (BMS) of 2.5, 2.75, or 3.0 mm × 12 mm (Medtronic Cardiovascular, Santa Rosa, CA) was deployed in the selected coronary segments. Stents were placed per standard interventional procedure in mid-LAD locations comparable ot those used in the MI model (see below), with a target stent to artery ratio of approximately 1.1–1.2:1.0 by online quantitative angiography.

### Model creation: MI animals

Using baseline angiography, a coronary angioplasty balloon was advanced to the mid-left LAD. The balloon was inflated to a balloon to artery diameter ratio of 1.2:1 for a total of 60 min to ensure total vascular occlusion. Balloon deflation was immediately followed by 15 min of reperfusion, during which a BMS of 2.5, 2.75, or 3.0 mm × 12 mm sizing (Medtronic Cardiovascular, Santa Rosa, CA) was deployed at the site of preceding occlusion to simulate stenting at the site of the culprit lesion responsible for the coronary occlusion causing the MI. Stents were placed per standard interventional procedure, with a target stent to artery ratio of approximately 1.1–1.2:1.0 by online quantitative angiography.

### Device description and SSO_2_ intracoronary infusion

The TherOx DownStream® System (TherOx Inc, Irvine, California), previously described^7,8^, was used to deliver the SSO_2_ infusion. The small extracorporeal circuit pumps blood from an arterial access site (5F sheath) and mixes it with a small amount of highly oxygenated saline (pO_2_ ~ 30,000 mmHg), producing a hyperoxemic perfusate (pO_2_ level of 760–1000 mmHg) at 5–7 × the normal oxygen concentration of blood plasma. This perfusate was infused into the LAD via a 4.6 French MI Cath Infusion Catheter (through the contralateral arterial access site (7F)). The procedure required a 5–10 min setup time and a one-time, 90-min infusion at a 50 ml/min flow rate. SSO_2_ infusion was started immediately after stent implantation. A normoxemic (O_2_ 150-200 mmHg) perfusate was delivered by the system to the control group. The infusion duration was 90 min in both groups.

### Angiography

At Day 0 and Day 7 or 30 angiograms were assessed for structural defects (*e.g.* dissections, ectasia), flow abnormalities, and thrombus presence. Coronary flow was assessed by TIMI flow grade^10^.

### Echocardiography

Two-dimensional echocardiography images for quantitative assessments were acquired in the right lateral decubitus position, using a 5-MHz probe (iE33, Philips Medical Systems, Bothell, WA) in the standard parasternal long-axis and short-axis views. Left ventricle (LV) end-diastolic diameter and end-systolic diameter were determined from short-axis planes; end-diastolic volume (EDV) and end-systolic volume (ESV) were calculated from long-axis planes. LV ejection fraction (EF) was calculated using a standard formula (EF = [(EDV − ESV) / EDV] × 100). LV septal and posterior wall thicknesses were measured at the mid-level from short-axis planes. Segmental wall motion scores were analyzed, per recommendation of the American Society of Echocardiography^11^, using a 16-segment model. Each segment was assigned a score based on its contractility: normal = 1, hypokinesis = 2, akinesis = 3, dyskinesis = 4. A wall motion score index (WMSI) was then calculated (WMSI = sum of wall motion scores / number of segments visualized). Since among all 16 segments of the whole heart most are normal and not affected by the myocardial infarction, a subset of segments adjacent to the infarcted area were also analyzed to determine a regional wall motion score (Anterior WMSI).

### Tissue harvest and histology processing

Hearts were removed immediately following pre-determined termination (7 ± 1 or 30 ± 2 days) and arteries were flushed with Ringer’s lactate for 15 min (1 L). Pressure perfusion fixation was performed for 20 min with 10% neutral buffered formalin. The fixed heart was immersed in a formalin filled container for at least 24 h to continue further fixation. The LAD was dissected and sectioned at proximal, mid, and distal locations of the stented segment. LAD samples were embedded in SPURR resin, sectioned at 5-micron intervals and stained with hematoxylin and eosin (H&E).

Hearts were sectioned across the baso-apical axis, from apex to base, into ~ 8 mm thick slices and examined. Non-MI model hearts were examined grossly and any gross abnormalities were photographed. The 5 apical most slices were placed whole (left and right ventricle) on their basal face in oversized cassettes, processed in paraffin, cut at 5 microns serially, and stained with H&E. MI hearts were examined macroscopically. Six myocardium slices were processed in paraffin, cut at 5 microns twice serially, and stained with H&E and Masson’s Trichrome for planimetric morphometry analysis and measurement of infarct size.

### Qualitative histology

All sections of myocardium were evaluated for thrombus, emboli (microemboli), inflammation, lymphohistocytic infiltrates, myocardial fibrosis (infarct scar) or other notable microscopic changes in the myocardium. For each parameter, score of 0 denoted absence, 1 = minimal presence, 2 = single focal presence, 3 = multifocal presence. In each animal, 7 myocardial regions were evaluated in each of the 6 slices for a total of 42 regions. Summary incidence values summed the scores attributed to each of the myocardial areas delineated in the data (left anterior, posterior, lateral ventricle and septum, and right anterior, posterior and lateral ventricle for a total of 7 general areas per tissue section at each of 6 sampling levels per heart equaling a total of 42 possible scoring entries per animal for each finding; with 5 animals in each group, there were 210 myocardial regions examined in each of the eight 5-animal subgroups. Summary values excluded early death animals.

To assess the biological and healing response of vascular tissue to BMS implantation and to identify any possible effects of treatment, ordinal data were collected, and a semi-quantitative scoring system was used to describe vascular tissue responses: peristrut inflammation vessel wall injury, neointima maturity, media hypocellularity, adventitial fibrosis, and endothelialization. Scores range from 0 to 3 (0 = feature absent/no change; 3 = overwhelmingly present feature or full neointima maturity or endothelialization). Specific morphologic and cellular features used to score each specific parameter are described in Online Resource 1.

### Quantitative histology of myocardium (MI model)

Infarct boundaries were traced and measured with Image Pro Plus (Media Cybernetics, Bethesda, Maryland). The following morphometric data were collected and analyzed: Histology Section Myocardium Area (HMA) (mm^2^), Total Histology Section Infarct Area (HIA) (mm^2^), Total Tissue Slice Area (TSA) (mm^2^).

From these direct measurements, all other histomorphometric parameters were calculated: Total Number of Infarcted Areas regardless of size, Histology Section Infarct Ratio (HIR): HIR = HIA/HMA; Minimum and Maximum Area (mm^2^) of infarction; Average Infarct Area (mm^2^): ΣIA/number of infarcted areas; Infarct Volume (IV) (mm^3^): IA*Tissue thickness (~ 8 mm); Total Infarct Volume (TIV) (mm^3^): TIV = ΣIV; Absolute Infarct Area (AIA) (mm^2^): AIA = TSA*HIR; Absolute Heart Infarct Volume (HIV) (mm^3^): HIV = Σ(AIA*Tissue thickness) (~ 8 mm); Total Myocardium Volume (TMV) (mm^3^): TMV = Σ(TSA*Tissue thickness) (~ 8 mm) and Infarct to Myocardium Ratio (IMR): IMR = HIV/TMV.

### Control of bias and statistical analysis

In general, animals were chosen for evaluation based on their Test Facility assigned identification number, in ascending order. This order was adjusted as required as a result of procedure cancellations or animal substitutions due to housing or health issues. Each animal received either test or sham infusion, per randomization (randomization order was determined prior to the study initiation using an online random number generator found online at www.randomizer.org). Due to the blinding of the echocardiography, clinical pathology and pathology analysts to the treatments, the targeted sequence of enrollment was not included in the protocol but in a separate document (enrollment matrix) accessible only by the principal investigator. TherOx system operators were informed of individual treatment assignment just prior to stent implant (*i.e.* post-myocardial infarction). The interventionalist could not be blinded to the study treatment because the TherOx system’s display screen and audible signal clearly indicated whether the infusion was hyperoxemic or normoxemic. The survival duration and infarct or non-infarct conditions were pre-assigned, and the interventions were guided by quantitative angiography, thus reducing operator bias. The principal investigator unblinded the echocardiography, clinical pathology and pathology analysts after completion of the raw data generation.

Summary data of continuous variables are presented as mean ± standard deviation. Summary ordinal data are presented as the median, with data variability represented by the 25th and 75th percentiles. Morphometry and microscopic data were statistically analyzed using appropriate 2-group procedures. A t-test and one-way ANOVA were performed to test the difference of outcomes. A *p* value of 0.05 or below was considered statistically significant.

## Results

### Procedural outcomes

No system failures for the DownStream System were experienced in the study. The infusion flow rate was 50 ml/min for each animal assigned to either SSO_2_ Therapy or Control (sham infusion). All 42 study animals received a complete 90-min. infusion. One infusion catheter was used in 40/42 (95.2%) cases and two infusion catheters were used in 2/42 (4.8%) cases. In each of the cases where multiple MI-Cath Infusion Catheters were used, no adverse effects were observed in the study animals and the 90-min infusions were completed.

No significant difference was witnessed in means of highest (196.4 ± 10.4 mmHg and 193.2 ± 11.3 mmHg) and lowest (186.1 ± 7.6 mmHg and 177.0 ± 9.4 mmHg) pO_2_ recordings at 30, 60 and 90 min during SSO_2_ or Sham LAD infusion.

### Clinical outcomes

Forty-three animals were enrolled, of which 3 died early, of causes believed to be unrelated to the SSO_2_ Therapy. One animal died during the infarct model creation and thus received no intracoronary infusion. Another 2 animals died on Day 1 (MI cohort) and Day 5 (non-MI cohort), respectively. Necropsy revealed that the failure to control bleeding from the femoral access site was the most likely cause of death. In both swine the ACT was very volatile and excessive amount of heparin was needed to keep it in the therapeutic range above 250 s. In the MI animal, the microscopic changes in the heart were consistent with terminal hypotension and/or hypovolemia in relation with procedural bleeding and MI model creation. There were no changes in the stented LAD either that would suggest the coronary cause for the early death of this animal. In the non-MI animal, there were no microscopic changes in the myocardium or LAD sections that would suggest the cardiac cause for the early death of this animal.

The remaining 40 animals completed the study as planned without any adverse cardiovascular events, non-cardiovascular health issues or relevant clinical pathology abnormalities. The mortality in the MI study cohort was 10%, which is low but expected given relatively small infarct sizes commensurate with 60 min ischemia period.

### Key myocardial outcomes

#### Echocardiography

As expected, the non-MI animals in the 7 days group and 30 days group had significantly higher EF and lower WMSI than animals who had MI induced. There was a slight trend toward improved left ventricular recovery by 7 days as evidenced by the regional anterior WMSI in the SSO_2_-treated MI animals and change in EF from post MI to 7 days follow up. This trend was more pronounced at the longer follow-up (30 days) where both EF as well as anterior WMSI differed between SSO_2_ and sham animals, respectively. None of these differences reached statistical significance (Table 1).

**Table 1:** Key echocardiographic outcomes in the MI subgroups.

	7 Days		30 Days	
	MI + SSO2 (N = 5)	MI + Sham (N = 5)	MI + SSO2 (N = 5)	MI + Sham (N = 5)
Baseline EF	58.1 ± 2.6%	55.9 ± 3.8%	56.2 ± 5.9%	54.6 ± 2.3%
Post-MI EF	34.5 ± 12.4%	36.1 ± 3.6%	38.2 ± 4.6%	35.3 ± 2.2%
Post-Infusion EF	38.9 ± 10.9%	38.8 ± 5.2%	41.4 ± 5.0%	36.5 ± 4.8%
Terminal EF	55.9 ± 2.5%	55.5 ± 4.0%	50.9 ± 4.0%	46.6 ± 1.9%
Terminal Anterior Wall Motion Score Index	1.30 ± 0.18	1.37 ± 0.46	1.40 ± 0.22	1.20 ± 0.21

EF, Ejection Fraction.

#### Myocardial histopathology

Myocardial histopathology findings of note are summarized in Table 2. Overall, of 210 regions evaluated in each of the eight 5-animal subgroups, majority of regions presented with no abnormal findings (score 0 for all parameters) except for infarct scars in the 4 MI subgroups, as expected of the model. When found, the abnormalities were mostly scored 1 (minimal presence) and rarely 2 (single focal presence).

**Table 2:** Summary of myocardial microscopic changes of note.

Microscopic finding/group	MI-SSO2	MI-Sham	Non-MI-SSO2	Non-MI-Sham	MI-SSO2	MI-Sham	Non-MI-SSO2	Non-MI-Sham
Time point (Days)	Day 7				Day 30			
Number of animals examined	5	5	5	5	5	5	5	5
**Mixed cell infiltrate**	**8**	**2**	**1**	**3**	**14**	**13**	**24**	**9**
Score 1	8	2	1	3	14	13	24	7
Score 2	0	0	0	0	0	0	0	2
**Infarct scar**	**66**	**76**	**2**	**2**	**32**	**53**	**5**	**4**
Score 1	43	57	2	1	29	49	5	4
Score 2	23	19	0	1	3	4	0	0
Number of animals with finding at any level	5	5	2	2	5	5	2	1
**Microgranuloma**	**0**	**3**	**0**	**1**	**9**	**5**	**1**	**3**
Score 1	0	3	0	1	9	5	1	3
**Foreign body microembolus**	**2**	**3**	**4**	**3**	**12**	**4**	**2**	**9**
Score 1	2	3	4	3	11	4	1	8
Score 2	0	0	0	0	1	0	1	1
**Depletion, myocardial fibers**	**2**	**4**	**10**	**4**	**21**	**7**	**5**	**8**
Score 1	2	4	8	4	21	7	4	7
Score 2	0	0	2	0	0	0	1	1
**Thrombus**	**0**	**0**	**0**	**0**	**0**	**0**	**0**	**0**
Total number of myocardial regions examined per animal	42	42	42	42	42	42	42	42
Total number of myocardial regions examined per group	210	210	210	210	210	210	210	210

The myocardium microscopic data demonstrated the expectedly high incidence of infarct scars in the MI treatment groups. The incidence of this finding was slightly reduced in the SSO_2_ treated groups compared to the time-matched Sham group. The magnitude of this decrease was slight on Day 7 and more definitive on Day 30 but not statistically significant at either study end point.

There was a low incidence of other background and procedure-related changes such as foreign body microemboli (particulate), microgranulomas and mixed cell infiltrates. These changes appeared at a low incidence in all groups with no evidence of direct relationship to treatment.

There were occasional small foci of myocardial depletion (micro-infarcts) that were related to minute distal ischemic events. Some of these lesions co-located with microemboli. It is likely that these foci were related in large part to particulate distal embolization. Some of the foci may also represent the attenuated expression of low-grade ischemia that may be related to model creation (infarction). However, the incidence or severity of this change did not show a relationship to model creation and/or treatment, except in SSO_2_ treated infarct group on Day 30. This group also showed the lowest incidence of overt infarct scarring. It is possible that the increase in myocardial depletion foci in this group represents the attenuated expression of overt infarct scars into a greater number of smaller microscopic areas of depletion, consistent with treatment efficacy.

Two animals in the 7-Day non-MI SSO_2_-treated group showed minimal and localized acute myocardial necrosis. The exact cause of this acute change was unclear. The morphology of the necrosis suggested a 2–3 day time course and therefore appeared unrelated to the procedure or SSO_2_ treatment.

**Figure 2:**
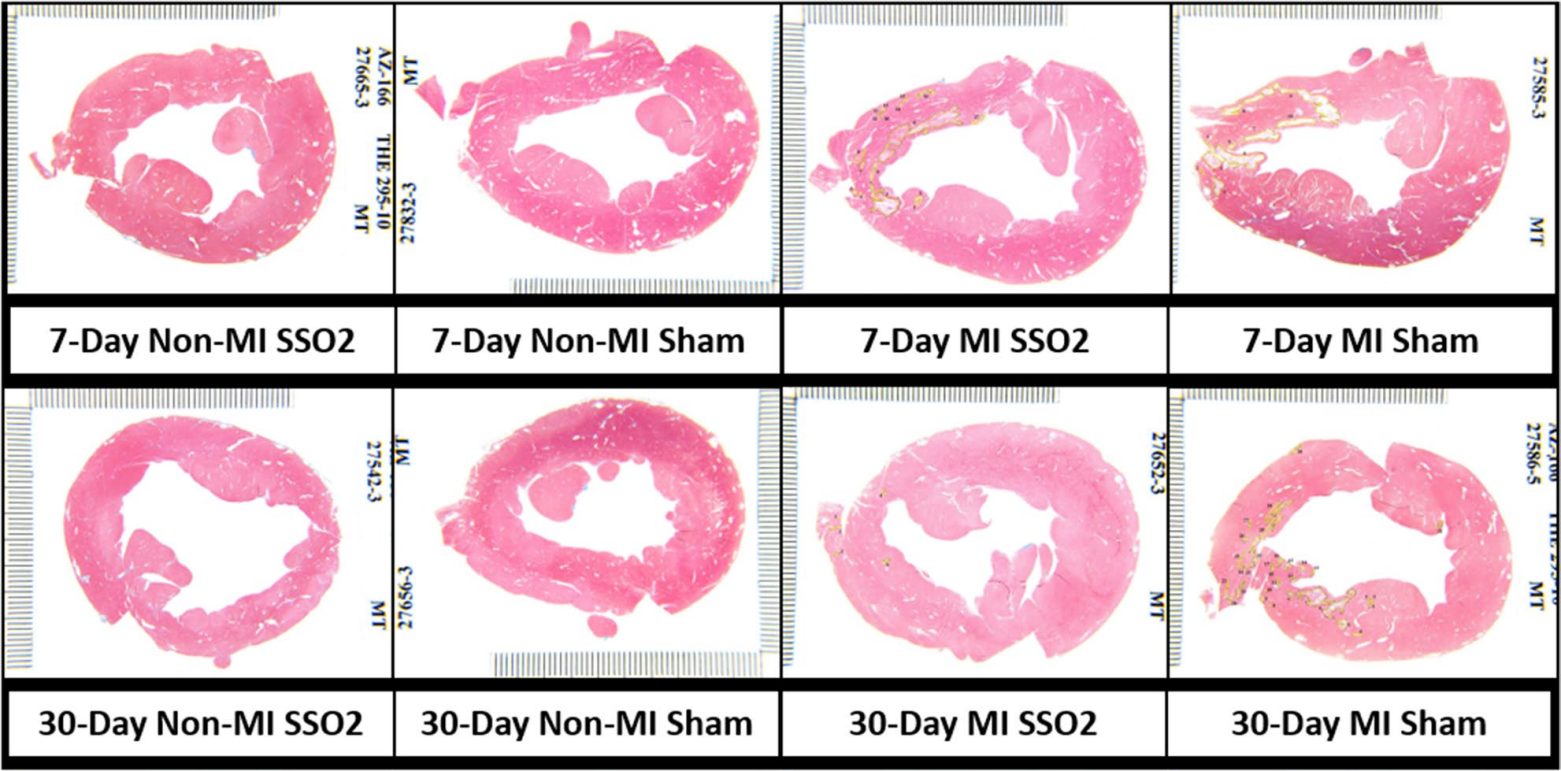
Side-by-side comparison of representative mid-basal left ventricular cross-sections from all 8 study subgroups. In the MI groups, areas of myocardial necrosis/scars, as defined by the study pathologist, are outlined by yellow lines.

Representative mid-basal left ventricle cross-sections from each of the 8 groups are shown in Fig. 2. The cross-sections from MI groups contain tracings of the infarcted areas and the number of identified infarcted areas in the particular cross-section.

#### Myocardial morphometry (infarcted hearts)

**Figure 3:**
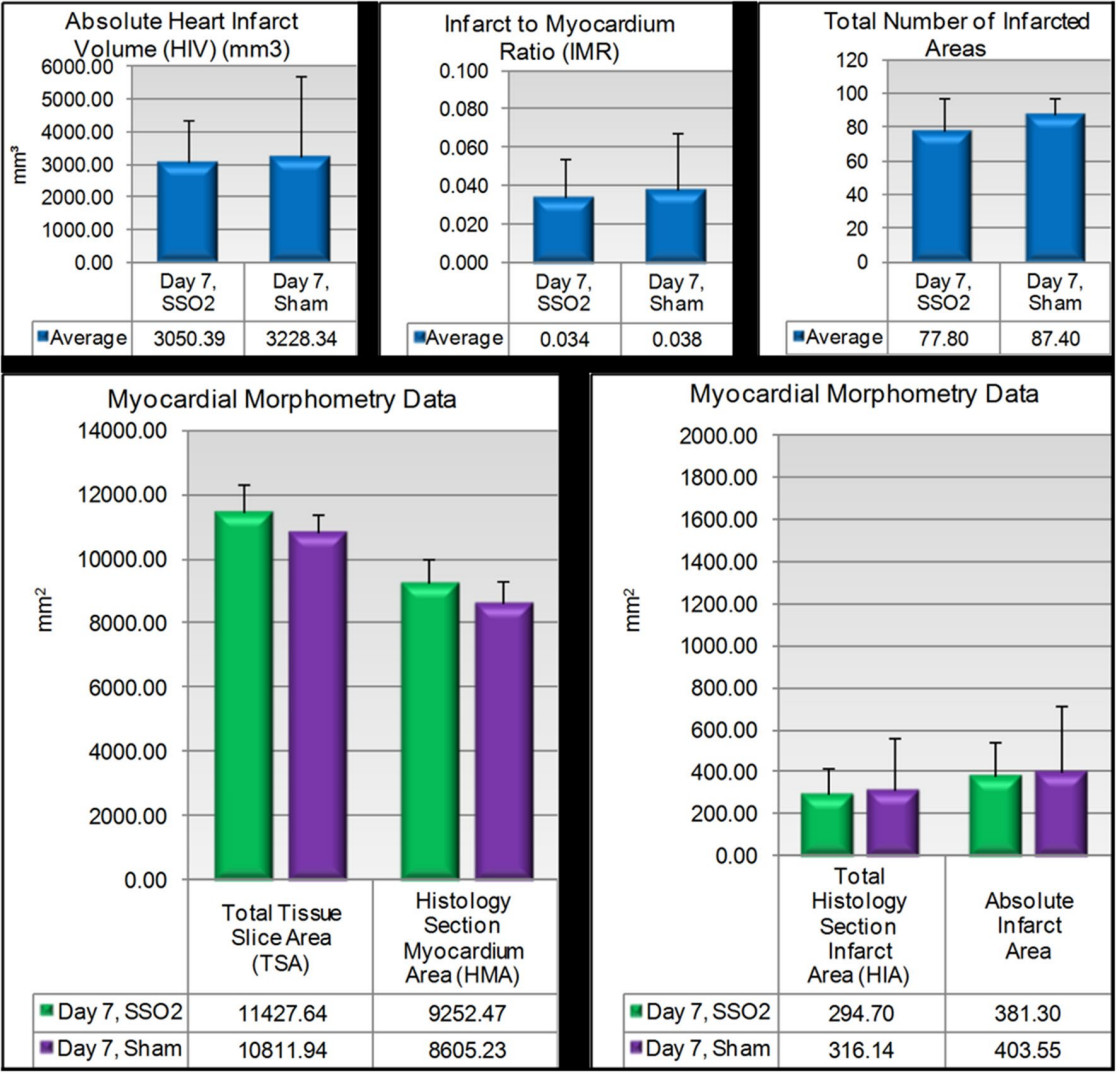
Key myocardial morphometric indices at 7 days. No early differences in histomorphometric indices of myocardial infarction injury can be observed hearts subjected to the SSO_2_ therapy in comparison to sham.

At Day 7, the myocardial morphometry data, graphically summarized in Fig. 3, showed an average infarct volume per heart of ~ 3.2cm^3^ in the Sham group and ~ 3.1cm^3^ in the SSO_2_ group (upper left panel). Infarct areas and volume, infarct-to-myocardium ratio, and the total number of infarcted areas were generally comparable between the Sham and SSO_2_ groups on Day 7. The total myocardium area measured on an identical number of section levels at comparable levels was slightly greater in the SSO_2_ group suggesting improved tissue viability.

**Figure 4:**
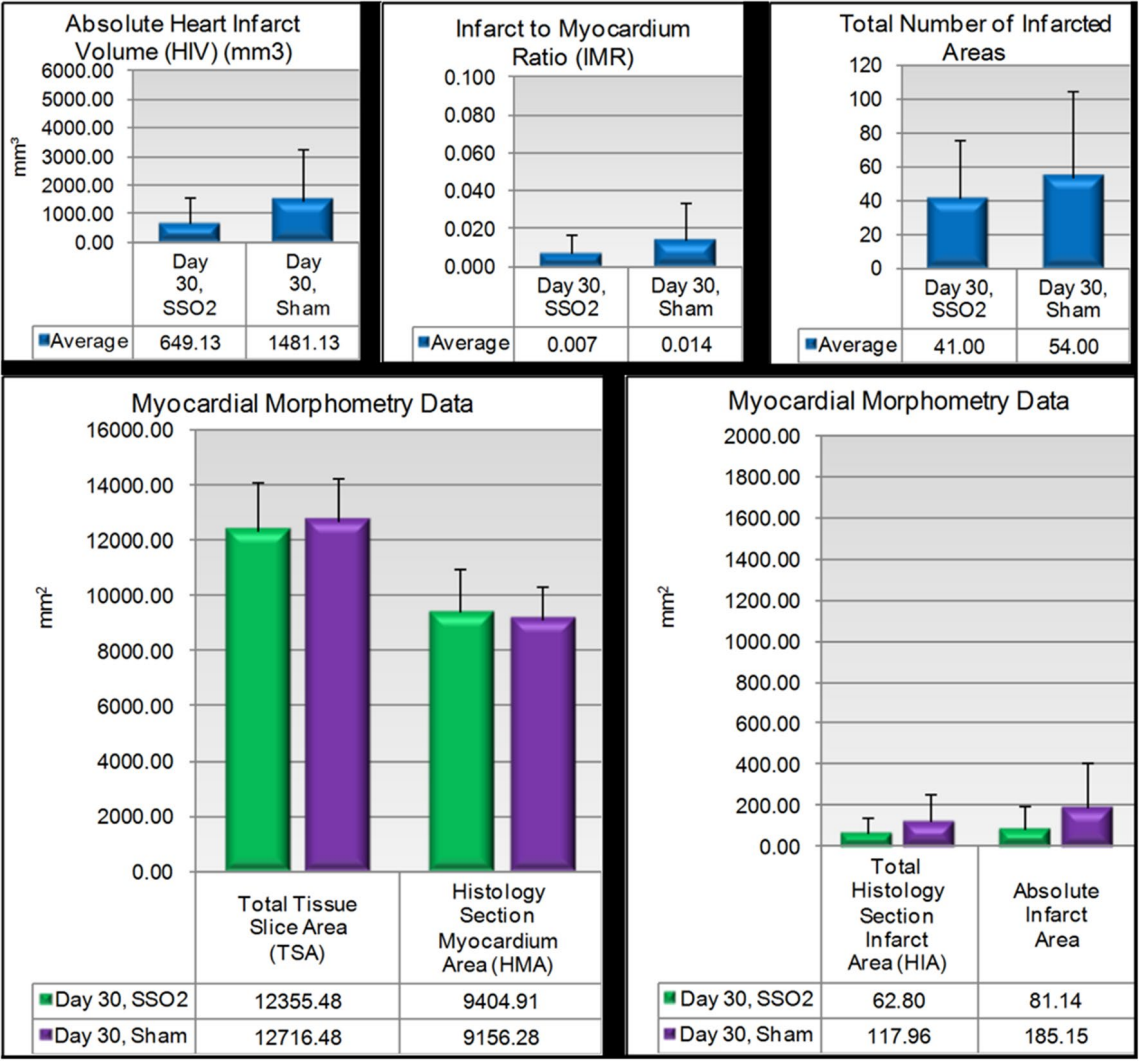
Key myocardial morphometric indices at 30 days. Note the trend toward less prominent histomorphometric indices of myocardial infarction injury in the SSO_2_ therapy in comparison to sham.

At Day 30, the myocardial morphometry data, graphically summarized in Fig. 4, showed an average infarct volume per heart in the Sham group of ~ 1.5cm^3^ per heart versus ~ 0.6cm^3^ in the SSO_2_ group. There was trend toward decrease in average total infarct volume and area, infarct-to-myocardium ratio, and the total number of infarcted areas in the SSO_2_ group compared to the Sham group, compatible with a slight increase in total myocardium area in the SSO_2_ group compared to the Sham-treated hearts. Overall these variations indicated a positive trend for SSO_2_ in reducing infarct size compared to Sham. There was clearly no increase in infarct areas in the SSO_2_ group under the conditions of this study.

### Key outcomes evaluation of stented arteries

#### Angiography

Qualitative angiography demonstrated no evidence of thrombus, dissection or ectasia in any animal either pre-treatment, post-stenting, post-infusion or terminal follow-up. As expected, the most common angiographic finding at follow-up was in-stent stenosis which was observed in 18/20 of the 30-day animals and none of the 7-day animals. There did not appear to be any consistent association of stent stenosis with treatment group. The visually estimated degree of stenosis in the 30-day animals is summarized by subgroup in Table 3. Post-infusion, TIMI flow values were all 3 in all 20 MI animals. TIMI flow grade 2 was reported in 8/20 non-MI animals post-infusion. These findings were thought to be related to the depth of anesthesia in a long procedure rather than the true flow impairment; although there was no formal angiographic quantification of myocardial blush of frame count, the angiographic analyst blinded to treatment did not report any observations suggestive of no-reflow or any other angiographically evident microvascular flow impairment. All 7-day and 30-day terminal angiographic examinations featured TIMI 3 flow grade and no other angiographically evident coronary flow abnormalities.

**Table 3:** Incidence of in-stent stenosis in 30-day animals.

Treatment group	Degree of in-stent stenosis by visual estimate			
	Mild	Moderate	Severe	None
MI + SSO2 (N = 5)	1	1	3	0
MI + Sham (N = 5)	1	1	3	0
Non-MI + SSO2 (N = 5)	2	0	2	1
Non-MI + Sham (N = 5)	2	1	1	1

#### Histopathology

**Figure 5:**
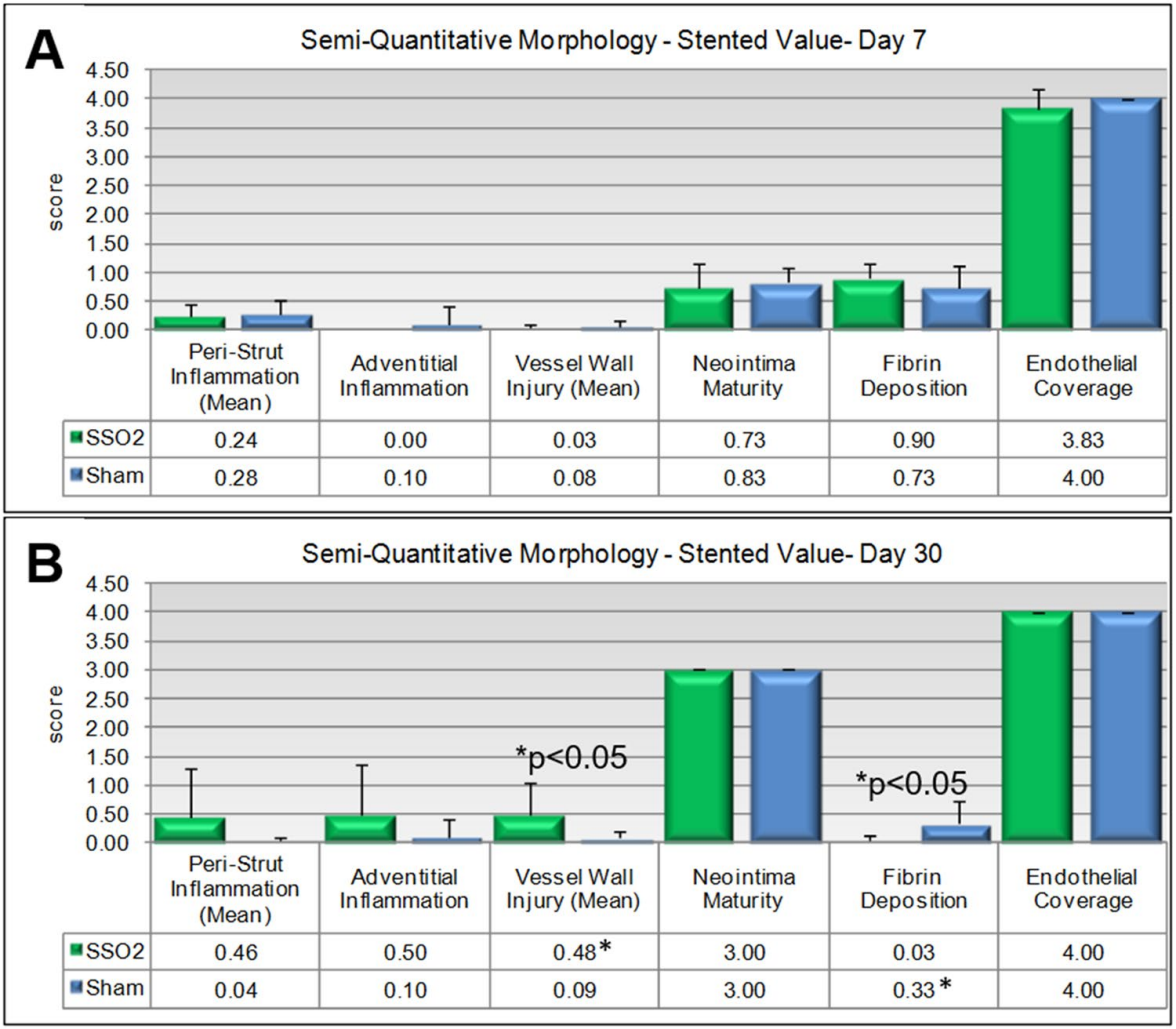
Key semiquantitative metrics of arterial healing at 7 days (**A**) and at 30 days (**B**). Overall, there is no negative impact of SSO_2_ therapy on the arterial healing parameters in comparison to sham. The incidental statistically significant differences in injury and fibrin deposition are discussed in the text.

**Figure 6:**
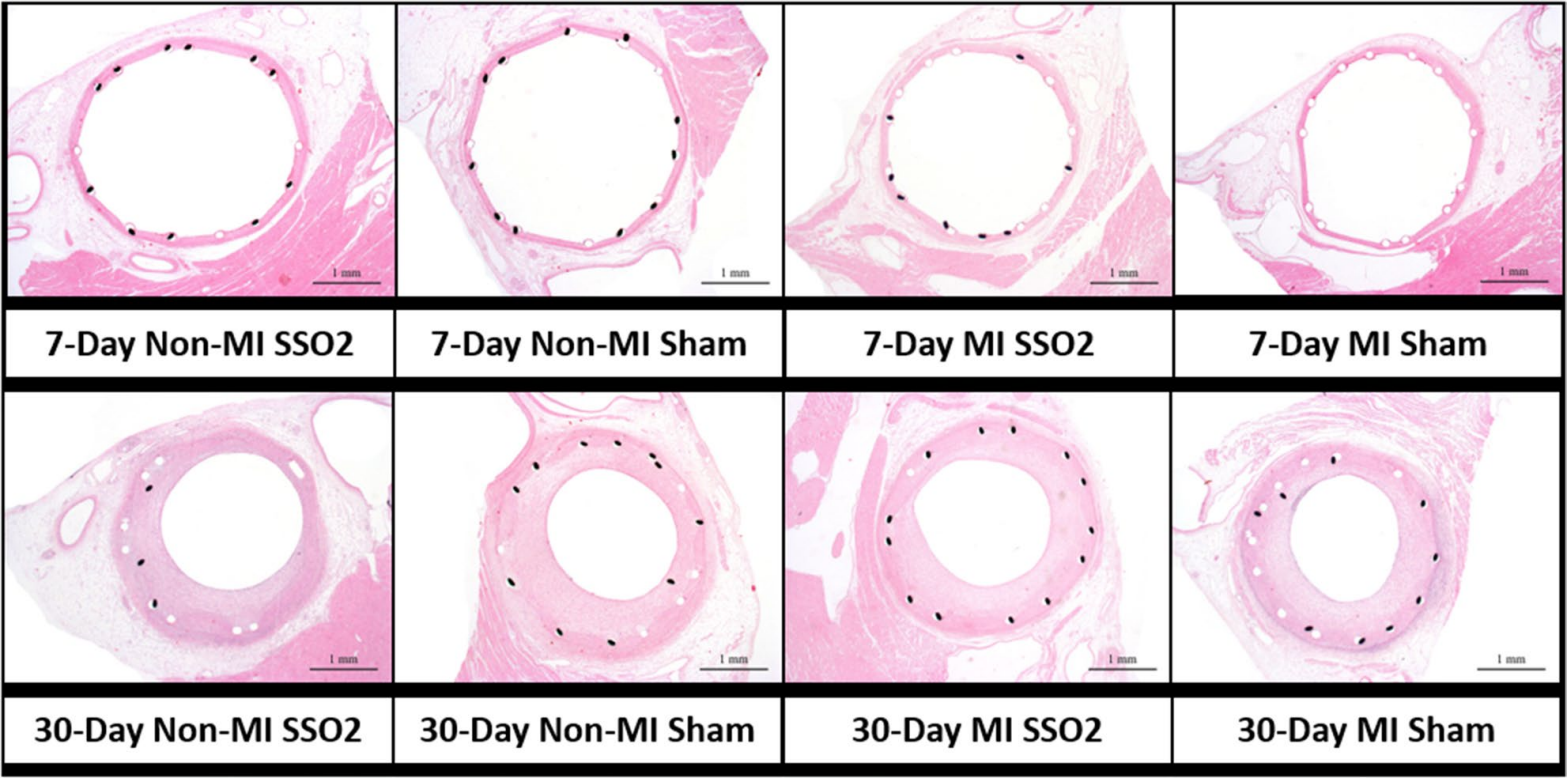
Representative in-stent coronary arterial cross-sections from all 8 study subgroups. Typical histological responses to stent placement in normal porcine arteries at 7 days (upper panels) and 30 days (lower) panels. No excess early thrombus formation at 7 days or excess neointima formation at 30 days are seen as a result of SSO_2_ therapy in comparison to sham. *Note*: Figs. 1, 2 and 6 generated with Microsoft Office 365 utilizing histology and histomorphometry images generated with ImagePro Plus (MediaCybernetics). Figs. 3, 4 and 5 generated with SigmaPlot v 11.0 (Build 11.2.0.5).

The presence or absence of MI had no impact on the arterial responses to stent placement so the results were only differentiated between SSO_2_ (n = 20) and Sham (n = 20) and presented in Fig. 5A (7-day outcomes) and Fig. 5B (30-day outcomes). Peri-strut inflammation was minimal and of comparable average score on Day 7 in both groups and appeared slightly increased in the SSO_2_ group on Day 30 compared to the Sham group. This apparent increase closely correlated with a matching increase in procedural injury that reached statistical significance, and with adventitial inflammation. These increases are believed to be related to variations in procedural injury at stent deployment and independent of SSO_2_ treatment. Other healing characteristics in the stented vessels in line with expectations after deployment of a bare metal stent and comparable in both the SSO_2_ and Sham groups. Namely, slight fibrin deposition was observed on Day 7 and was largely resolved at 30 days. Maturation of the neointima progressed comparably in both groups and the neointima was fully mature at 30 days in both groups. The minimal, yet statistically significant increase in neointima fibrin in the sham group on Day 30 compared to the SSO_2_ group was not considered to be biologically or toxicologically meaningful. Endothelialization was complete in both groups at 30 days. At 7 days there were a few vessels with incomplete endothelialization, namely over areas of unresorbed fibrin. This change was expected at 7 days and due to the very small magnitude of the decrease in the treated group, the variation was considered unrelated to SSO_2_ treatment. Examination of proximal and distal unstented LAD coronary vessels showed no microscopic changes specifically attributable to SSO_2_ treatment. Overall, there were no unexpected changes and no features that suggested an effect of SSO_2_ treatment on vascular healing at 7 or 30 days. Representative cross-sections from each group are presented in Fig. 6.

## Discussion

SSO_2_ therapy has emerged in the last decade as possible augmentation for revascularization in MI patients and it has been approved for clinical use in the US. Both animal as well as clinical studies were able to show different degrees of efficacy^1,12,13^. However, data providing histopathologic insight into of both normal and infarcted myocardium subjected to SSO_2_ therapy with a commercially established system, as well as histologic assessment of the “culprit” coronary artery, i. e. the infarct-related artery undergoing percutaneous recanalization followed by stent placement, have not been presented to date.

Accordingly, we report here an animal study designed to comprehensively evaluate safety and feasibility aspects of the SSO_2_ delivery procedure in healthy and infarcted domestic swine. We were able to show absence of SSO_2_ therapy related adverse effects on normal and non-infarcted myocardium, as well as on the stented coronary arteries. We also found a trend towards decline in MI volume and area in animals treated with SSO_2_ as compared to sham controls, assessed by histological examination 30 days following MI.

The acute effects of SSO_2_ therapy on ventricular function and infarct size in MI animal models have been previously studied and documented^5,6,14,15^. Following equivocal results of efficacy seen with the Acute Myocardial Infarction with Hyperoxemic Therapy (AMIHOT-I) trial, post hoc subgroup analysis of the study suggested that hyperoxemic reperfusion may reduce infarct size in patients with anterior ST-segment elevation MI treated within 6 h of symptom onset^16^. The follow-up AMIHOT II trial was performed and utilized a prespecified Bayesian approach to allow partial pooling of evidence from the AMIHOT-I trial to be incorporated into the analysis of data in the AMIHOT-II trial. AMIHOT II was a positive trial and met its pre-specified endpoint for infarct size reduction with SSO_2_ infusion as compared to controls^9^. Report by Hanson et al.^13^ described the safety and feasibility of a modified delivery system and technique using SSO_2_ in patients treated with PCI within 6 h of anterior STEMI. This was further validated in the single-arm IC-HOT study^17^, with improved 1-year clinical outcomes including lower rates of death and new-onset heart failure or heart failure hospitalizations when compared with a propensity-matched control group^18^.

The animal model has inherent limitations when it comes to evaluating device efficacy but serves as an excellent candidate for safety evaluations^19^. Similar to previous studies, we witnessed improvement in ventricular function by echocardiographic measurements in the group treated with SSO_2_ compared to the sham controls. Increase in EF together with decrease in anterior WMSI were recorded at 7 days and were only slightly better in the SSO_2_ group. The difference between the two groups became more prominent at 30 days, although without reaching statistical significance. WMSI has been reported to be a more accurate method of assessing LV functions after reperfusion because it negates the effect of non-infarct zone hyperkinesis^8,20^. A regional wall motion score was determined since most of the 16 segments of the whole heart were normal, hence the need to analyze a subset of the segments regionally which more accurately represents the infracted area. Though no significant difference was seen in either therapy, the trends of both the %EF and Anterior WMSI might imply better prognosis for left ventricular function with SSO_2_ therapy.

One of the animal model’s advantages is the ability to evaluate tissue effect of therapeutic interventions by means of direct histological exam rather than by surrogate imaging modalities. Overall, there no adverse effects attributable to SSO_2_ delivery were observed on myocardium of non-infarcted swine, nor on the non-infarcted myocardium in the MI swine. We observed a difference in infarct volume and areas as measured by histology, showing smaller infarcts for the SSO_2_ treated animals. Once again, the effect of treatment was modest after 7 days but seen clearly at the 30 days end point, without reaching statistical significance. Histological morphometric analysis showed an increase in myocardial tissue area and volume in the SSO_2_ group at both time points, suggesting improved viability of myocardial tissue compared to the control group. These results are in agreement with previous report by Spears et al.^5^ further emphasizing the favorable effect SSO_2_ has on the tissue level.

Concerning safety of the use of SSO_2_ in regard to the coronary vasculature, histological examination showed no adverse changes in stented LAD arteries and adjacent non-stented segments as a result of SSO_2_ treatment. The stented vessels showed expected healing and biocompatibility changes for this type of procedure and implantation with a bare metal stent, including variable degree of neointimal formation/in-stent stenosis commensurate with mechanical injury inflicted by the stent as it typical in the porcine model in absence of atherosclerosis. Although at 30 days, the injury score and the fibrin score were statistically significantly different between SSO_2_ and Sham, the average scores were very low in both groups (less than 0.5 on a 0–3 scale) and the fibrin trended in the opposite direction as injury and inflammation. In genuinely poorer healing, all these parameters would trend in the same direction and the scores would be at least above 1.5 on average. Thus, these differences were considered serendipitous and not related to treatment. No evidence of thrombus was noted in any stented vessels for any animals treated with either SSO_2_ or Sham treatment, regardless if MI was induced or not.

Evaluation of the downstream organs did not show any adverse effect such as infarcts or thromboemboli. Notwithstanding, two out of three deaths during the study were considered to be related to periprocedural bleeding. Concern regarding periprocedural bleeding was raised following the clinical trials evaluating SSO_2_ as well^13^. It is because of that the manufacturer made changes in the delivery system and technique including the use smaller vascular sheath and minimizing the number of arterial punctures needed to complete the procedure^13^.

Several speculations were made trying to shed light on the pathway by which SSO_2_ influences cardiac tissue including inhibition of leukocyte adhesions^21^, alteration of nitric oxide synthase expression^22^, quenching of lipid peroxidation^23^, downregulation of β2 integrin^24^, and reduction of capillary endothelial cell swelling^25^. As a debate continues about the exact role of oxygen in the treatment of patients with MI, and some evidence suggests even harmful role for it in normoxemic patients^1,12^, it is important to differentiate that from the use of supersaturated oxygen as a therapeutic modality. Our study design was planned to assess safety and as such was not powered for efficacy measurements. As such, the model was designed for survival and not maximum infarction. Nevertheless, the trends of improvement seen in both functional and histological measurements, add in our opinion to growing body of evidence suggesting efficacy of such treatment in the population of patients with large anterior myocardial infarctions. More importantly, absence of adverse effects on normal myocardium and the arterial response to stent placement is reassuring in view of earlier concerns that SSO_2_ therapy may be damaging to the myocardium via abundant generation of oxygen free radicals.

Several limitations of this investigation should be acknowledged. The study design was a compromise between the regulatory requirements the study aimed to satisfy, the very complex logistics to complete the study in a reasonably short time, and the high cost associated with this endeavor. . The small sample size of n = 5 in each of the 8 treatment arms was therefore arbitrarily decided as minimally adequate based on historic data. This decision naturally underpowered analysis, not allowing the positive trends becoming significant. Although the study was not designed to be powered for efficacy measurements but rather for proving noninferiority in safety aspects, the trend of a higher ventricular recovery in the SSO_2_ groups is notable. Another possible limitation in the model design is the use of an infarct model of 60 min rather than longer, which let to modest infarct sizes. On the background of modestly sized control infarcts, demonstrating statistically significant improvements by the therapy is more challenging. Freyman et al.^26^ reported that similar technique used in their lab resulted in an infarction affecting 20–30% of the left ventricular wall mass. Also, the study did not include an 24-h time point to assess the early response but relied on historical data in that regard^6^. Lastly, the study used an early delivery technique that does not include all later modifications discussed above, reported in more recent studies^13,17,18^ and presently used clinically.

## Conclusion

In both normal and anterior MI porcine models, intracoronary treatment with hyperoxemic SSO_2_ therapy showed no evidence of coronary thrombosis or any toxicity (arterial, distal myocardial or systemic) after 7 or 30 days. A trend was observed toward reduced infarct size in histopathology, as well as toward better recovery of echocardiographically evaluated global and regional contractility at 30 days.

## Supplementary Information

Supplementary Information.

## Data Availability

The datasets generated during and/or analyzed during the current study are not publicly available due to their proprietary nature (owned by the study sponsor) but are available from the corresponding author on reasonable request.

## Abbreviations

SSO_2_: Supersaturated oxygen
MI: Myocardial infarction
EF: Ejection fraction
WMSI: Wall motion score index
PCI: Percutaneous coronary intervention
TIMI: Thrombolysis in Myocardial Infarction
TTE: Transthoracic echocardiograms
BMS: Bare metal stent
LAD: Left anterior descending coronary artery
EDV: End-diastolic volume
ESV: End-systolic volume
H&E: Hematoxylin and eosin
