# Supplementary material for: Chronic myocardial and coronary arterial effects of intracoronary supersaturated oxygen therapy in swine with normal and ischemic-reperfused myocardium
Source: Sci Rep. 2022 Apr 6;12:5785. doi: 10.1038/s41598-022-09776-8 (PMC8987078; doi:10.1038/s41598-022-09776-8)

**Kaluza GL et al.** **Chronic Myocardial and Coronary Arterial Effects of Intracoronary Supersaturated Oxygen Therapy in Swine with Normal and Ischemic-Reperfused Myocardium.**

**ONLINE RESOURCE 1: Details of the semiquantitative scoring of the coronary arterial histology sections.**

A semi-quantitative analysis was performed to assess the biological and healing response of vascular tissue to the stents and identify any possible effect of treatment. Specific morphologic and cellular features used to score each specific parameter are described below:

Inflammation was scored on a per-strut basis. Degree score: 0=No change; 1=Present, but minimal feature, scant inflammatory cells not forming a solid rim around the strut; 2=Notable feature, solid rim of inflammatory cells not effacing the neointima, media and/or adventitia; 3=Overwhelming feature, thick rim of inflammatory cells effacing the vessel wall around the strut and forming a microgranuloma.

Vessel wall injury was scored on a per-strut basis. Score: 0=Not present; 1=Break in IEL; 2=Break in media; 3=Break in adventitia.

Neointima fibrin/fibrinoid deposits degree score: 0=Not present; 1=Light spotting of fibrin; 2=Heavier deposition of fibrin; 3=Heavy deposition of fibrin with linking between struts.

Neointima maturity = Neointimal smooth muscle cells score: 0=Not present; 1=Light dispersed smooth muscle population; 2=Heavier proportion of smooth muscle cells in the neointima with residual areas that are not fully mature, containing residual fibrin and/or granulation tissue; 3=Full neointima maturity with dense population of smooth muscle cells throughout the neointima.

Media hypocellularity degree score: 0=Not present; 1=Light subintimal areas of dispersed media hypocellularity; 2=Moderate and transmural media hypocellularity; 3=Marked media hypocellularity occupying wide media areas.

Adventitial fibrosis degree score: 0=Not present; 1=slight thickening of the adventitia focally or diffusely by adventitial fibrosis; 2=Moderate and thicker adventitial fibrosis; 3=Marked thickening of the adventitia circumferentially or over a large portion of the adventitia.

**ONLINE RESOURCE 2: Angiographic illustration of a representative case of occlusion balloon placement followed by the stent deployment and infusion catheter placement in the LAD coronary artery.**


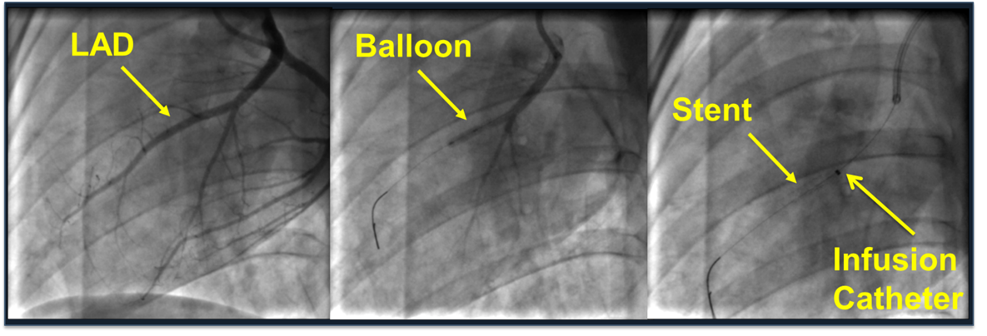

Supplement: Supplementary file 1 — Supplementary Information. [file 41598_2022_9776_MOESM1_ESM.docx]
